# Insights into the Structure and Function of the Pex1/Pex6 AAA-ATPase in Peroxisome Homeostasis

**DOI:** 10.3390/cells11132067

**Published:** 2022-06-29

**Authors:** Ryan M. Judy, Connor J. Sheedy, Brooke M. Gardner

**Affiliations:** Department of Molecular, Cellular, and Developmental Biology, University of California, Santa Barbara, CA 93106, USA; ryanjudy4@gmail.com (R.M.J.); connorsheedy@ucsb.edu (C.J.S.)

**Keywords:** peroxisomes, organelle biogenesis, AAA-ATPase, translocation, PEX1, PEX6, PEX26

## Abstract

The AAA-ATPases Pex1 and Pex6 are required for the formation and maintenance of peroxisomes, membrane-bound organelles that harbor enzymes for specialized metabolism. Together, Pex1 and Pex6 form a heterohexameric AAA-ATPase capable of unfolding substrate proteins via processive threading through a central pore. Here, we review the proposed roles for Pex1/Pex6 in peroxisome biogenesis and degradation, discussing how the unfolding of potential substrates contributes to peroxisome homeostasis. We also consider how advances in cryo-EM, computational structure prediction, and mechanisms of related ATPases are improving our understanding of how Pex1/Pex6 converts ATP hydrolysis into mechanical force. Since mutations in *PEX1* and *PEX6* cause the majority of known cases of peroxisome biogenesis disorders such as Zellweger syndrome, insights into Pex1/Pex6 structure and function are important for understanding peroxisomes in human health and disease.

## 1. Introduction

Peroxisomes are functionally diverse organelles ubiquitous across eukaryotes [1,2]. The single membrane of a peroxisome, populated by peroxisome membrane proteins (PMPs), surrounds a peroxisome matrix, which harbors enzymes that carry out specialized reactions, including the β-oxidation of fatty acids and the detoxification of reactive oxygen species [3]. Peroxisome matrix proteins are imported from the cytosol post-translationally, a process mediated by peroxisome biogenesis factors, the Pex proteins [4]. Peroxisome biogenesis and degradation are responsive to cellular conditions; the size, number, and function of peroxisomes depends on the organism, cell type, and a cell’s fluctuating metabolic requirements [5,6,7,8].

In this review, we focus on the heterohexameric ATPase complex Pex1/Pex6 and its partner protein—named Pex15 in *S. cerevisiae* or PEX26 in other organisms—that recruits Pex1/Pex6 to the peroxisome membrane [9,10,11,12,13]. Pex1 and Pex6 are ATPases associated with diverse cellular activities (AAA-ATPases) and are essential for peroxisome biogenesis and maintenance; as the only energy-utilizing Pex proteins, Pex1 and Pex6 drive the peroxisomal import of matrix enzymes and actively prevent peroxisome degradation [14,15,16,17,18].

In humans, mutations causing dysfunction in PEX1, PEX6, and PEX26—the human equivalents to yeast Pex1, Pex6, and Pex15—are the most prevalent cause of rare genetic disorders called peroxisome biogenesis disorders (PBDs) [19]. The severity of these disorders depends on the degree of peroxisome impairment. Complete peroxisome loss causes a lethal developmental disorder, Zellweger syndrome, while milder dysfunction can cause vision and hearing loss, fingernail and enamel abnormalities, and varying neurological defects [20]. As integral contributors to peroxisome homeostasis and human health, PEX1 and PEX6 are important research targets.

Although Pex1/Pex6’s mechanism, substrates, and functions are still incompletely understood, recent experiments, structures, and research on related ATPases are clarifying long-standing mysteries and redefining the important questions. After briefly describing the prevailing models for peroxisome biogenesis and matrix protein import, we review what is known about Pex1/Pex6, emphasizing how its structure informs discussions of its mechanisms and possible substrates. We conclude by considering how the most common pathogenic mutation in *PEX1*, resulting in PEX1-G843D, affects the motor’s function.

## 2. Peroxisome Membrane Biogenesis

**Figure 1:**
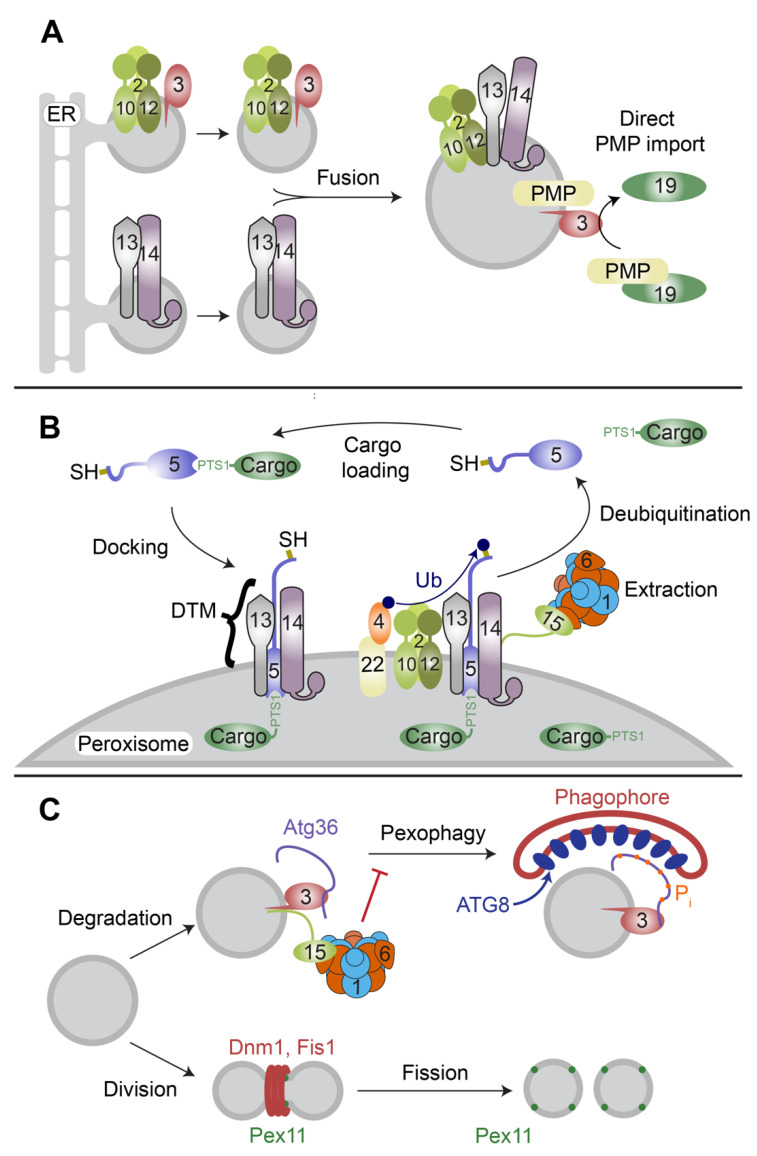
Overview of *S. cerevisiae* peroxisome biogenesis. (**A**) In de novo biogenesis, pre-peroxisomal vesicles (ppVs) carrying peroxisome membrane proteins bud and fuse with other ppVs or with mature peroxisomes. PMPs can also be directly inserted into the peroxisomal membrane by Pex19 and Pex3. (**B**) Pex5 binds the C-terminal PTS1-targeting signal on a cargo protein and interacts with the docking and translocation module (DTM) to import the cargo protein into the peroxisome. Pex5 is subsequently ubiquitinated by Pex4 and the Pex2/Pex10/Pex12 complex. Pex1/Pex6 extracts Pex5 from the peroxisomal membrane. Following deubiquitination, Pex5 can repeat the import cycle. (**C**) Mature peroxisomes can either grow and divide using the peroxisomal fission factor Pex11 and shared mitochondrial factors (Dnm1 and Fis1) or undergo peroxisome-specific autophagy mediated by adaptors such as Atg36. Pex1/Pex6 interacts with Atg36 through Pex3 and Pex15 to suppress Atg36 phosphorylation and pexophagy. Accessory proteins involved in process are represented as numbered proteins.

Peroxisomes can be formed by two overlapping pathways: de novo biogenesis by budding and fusion of pre-peroxisomal vesicles (ppVs) (Figure 1A), or by the growth and division of existing peroxisomes [6,21,22,23]. To generate peroxisomes de novo in yeast, a subset of PMPs is inserted into the endoplasmic reticulum (ER) using traditional insertion machinery, including Sec61 and the GET complex [24,25,26]. PMPs cluster into multiple distinct subdomains of the ER and bud to form ppVs [23,27,28]. After budding, ppVs are thought to heterotypically fuse to form the active import complex for the direct targeting of matrix and membrane proteins [29] to make mature peroxisomes. Mammalian cells undergoing de novo peroxisome biogenesis similarly generate distinct ppV populations by trafficking a set of PMPs to the ER and a distinct set to the mitochondrial outer membrane [30].

To grow and divide, mature peroxisomes require membrane lipids and PMPs. These can be obtained by fusion of mature peroxisomes with ppVs [30,31,32]; alternatively, peroxisomes can obtain lipids from the ER by direct peroxisome–ER contact [33] and PMPs by post-translational import from the cytosol [34]. Most PMPs harbor a helical-targeting signal—called a membrane peroxisome-targeting signal (mPTS)—near their transmembrane domains that serves as a recognition site for the cytosolic PMP receptor, Pex19 [35,36]. Pex19 shuttles between the cytosol and peroxisome membrane, shielding client transmembrane domains from aggregation and delivering them to a docking protein at the peroxisome membrane, Pex3 [37,38,39,40,41,42]. Pex3 and Pex19, perhaps with other unidentified factors, insert client proteins, including single-pass, multi-pass, and tail-anchored membrane proteins [43,44] (Figure 1A).

After sufficient growth, peroxisomes use Pex11 and mitochondrial fission machinery, including Dnm1 and Fis1, to elongate and divide [45,46,47,48,49]. Peroxisomes are degraded in response to nitrogen starvation, dysfunctional matrix protein import, or other signals using a peroxisome-specific macroautophagy called pexophagy [50] (Figure 1C).

## 3. Matrix Protein Import

Peroxisomal matrix enzymes are encoded by nuclear genes and are targeted for post-translational import into the peroxisome matrix by one of two peroxisome-targeting signals, PTS1 or PTS2. Amazingly, the imported proteins remain fully folded or even oligomerized as they are translocated [15,51,52], yet the membrane remains impermeable to molecules larger than about 600 daltons [53,54]. Most matrix proteins use a C-terminal peroxisome-targeting signal (PTS1) that is defined by the consensus sequence [S/A/C] [K/R/H] [L/H] and is sufficient to artificially target cytosolic proteins to the peroxisome [55,56]. PTS1 proteins are recognized by the soluble cytosolic receptor Pex5, which contains a C-terminal PTS1-binding domain and an intrinsically disordered N-terminal domain [57,58,59,60,61] (Figure 1B). Some cargo proteins lacking a PTS1 tag bind Pex5’s N-terminal domain; for these proteins, the N-terminal half of Pex5 is sufficient for import [62,63,64]. Cargo-bound Pex5 docks to its peroxisome membrane receptors, Pex13 and Pex14, to deliver cargo across the membrane by forming a pore-like structure called the docking/translocation module (DTM) [65,66,67,68,69] (Figure 1B). The minimal and actual DTM composition depends on the substrate and possibly the stage of import [70,71,72,73,74], but it typically comprises a hetero-oligomer of Pex5, Pex13, and Pex14 (with Pex17 in yeast) that changes in size depending on the size of the cargo [65]. Pex5’s N-terminal domain binds N- and C-terminal domains of Pex14 [66,75,76] and a C-terminal SH3 domain in Pex13 [68,77]. Although the membrane topology of Pex13 and Pex14 has been controversial [68,78,79,80], recent evidence suggests that Pex13’s N-terminus and Pex14’s C-terminus are in the peroxisome lumen, and the opposite termini are cytosolic [81,82]. Multiple copies of Pex5 oligomerize in the pore and these are thought to be integral structural components of the DTM [65], although the nature and relevance of any direct contacts between Pex5 and the lipid bilayer are unclear [61,67,83]. Pex proteins involved in Pex5 export—Pex8, Pex2, Pex10, Pex12, Pex15, Pex1, and Pex6—are also associated with the DTM [84].

Instead of a PTS1 tag, some peroxisome matrix proteins rely on a PTS2 tag near the N-terminus that is recognized by the globular protein Pex7 instead of Pex5 [85,86,87,88]. Pex7 requires a co-receptor to shuttle proteins across the peroxisome membrane. The co-receptor in mammals is a splice variant of PEX5, PEX5L, that differs from PEX5 only in the insertion of a PEX7-binding motif within PEX5’s N-terminal domain [89,90]. Similarly, yeast use the co-receptors Pex18 or Pex21, which resemble the N-terminal domain of Pex5 in domain architecture and function, but each contain a Pex7-binding domain in place of a PTS1-binding domain [62,91,92]. In each case, the co-receptor is incorporated into the DTM [74,93].

After embedding in the membrane, Pex5 or Pex7 must release their cargo into the matrix, but the mechanism of release is unclear, especially given the high local concentration of PTS1 tags. In one model, components of the DTM compete with the cargo for binding to Pex5 or Pex7. Pex13, Pex14, and Pex8 all bind Pex5 and each has been proposed to promote cargo release [69,71,94,95,96]. Presumably, a mechanism would have evolved to release cargo from each known binding site on the receptor: Pex5’s C-terminal domain, Pex5’s N-terminal domain, and Pex7. In an alternative model, receptor unfolding during extraction causes it to release its substrate into the matrix [97]. This model is supported by the recent finding that Pex5 is indeed globally unfolded during extraction [82,98].

Pex5 or the PTS2 co-receptor [99,100] is mono-ubiquitinated after embedding in the membrane, which is thought to be the signal for extraction [99,100,101,102,103] (Figure 1B). The peroxisome membrane-bound E3 ubiquitin ligase Pex12 [104] transfers a ubiquitin from an E2, Pex4 (UbcH5a, UbcH5b and UbcH5c in mammals), to a conserved cysteine near the N-terminus of Pex5 [105,106,107,108]. Cysteines are rare targets for ubiquitination and produce thioester-linked ubiquitin, which is less stable than the isopeptide bonds in lysine ubiquitination [109]. Some evidence suggests that cysteine ubiquitination allows Pex5 to act as a redox sensor, wherein cytosolic oxidative stress prevents Pex5 ubiquitination and thus slows peroxisome import [110,111]. Cargo with a low-affinity variant of a PTS1 tag, namely catalase, is particularly affected by the reduced import and remains in the cytosol, where it protects against oxidative stress [110,112,113]. After ubiquitination, Pex5 is extracted from the peroxisome membrane in a process driven by ATP hydrolysis in the AAA-ATPase Pex1/Pex6 [102]. This energy-dependent extraction is thought to provide directionality for the cycle, a concept termed “export-driven import” [114]. Finally, cytosolic Ubp15 (Usp9X in mammals) completes the cycle by deubiquitinating Pex5 [115,116], allowing recycling of Pex5 and co-receptors for multiple rounds of import.

## 4. Proposed Roles for Pex1 and Pex6 in Peroxisome Biogenesis and Maintenance

Pex1 and Pex6 assemble into a single heterohexameric motor belonging to a clade of type II AAA-ATPases with diverse cellular functions, including vesicle fusion, protein degradation, cytoskeleton remodeling, ribosome biogenesis, and protein extraction from membranes [9,11,117,118,119,120,121]. Pex1 and Pex6 were originally identified as genes necessary for yeast to grow on oleate, which requires peroxisomes, and were subsequently recognized as dysfunctional in humans with certain peroxisome biogenesis disorders [122,123,124,125,126,127]. Homology detection quickly revealed that Pex1 and Pex6 were closely related to several well-studied AAA-ATPases, notably N-ethylmaleimide-sensitive fusion protein (NSF) and Cdc48 (called valosin-containing protein or p97 in humans). NSF acts to dissociate post-fusion oligomers of soluble NSF attachment protein receptors (SNAREs) that form during vesicle fusion [117]. Cdc48 is a multifunctional unfoldase that uses a variety of partners to engage and unfold ubiquitinated substrates. Cdc48 can disassemble complexes, unfold proteins prior to proteasomal degradation, or extract proteins from membranes [121,128]. With these homologs as models, researchers identified two possible roles for Pex1 and Pex6: priming ppVs for fusion (analogous to NSF [129,130]) or extracting Pex5 from the DTM (analogous to Cdc48 in ER-associated degradation), which was known to be the energy-dependent step in matrix protein import [14,131] (Figure 1B). More recent research has contradicted the early conclusion that Pex1/Pex6 is necessary to prime ppVs before fusion [31,32], but has confirmed that Pex1/Pex6 is necessary to extract Pex5 from the peroxisome membrane for subsequent rounds of import [15,98,102]. Receptor recycling remains the canonical role for Pex1/Pex6 across eukaryotes.

In both mammals and yeast, cells deficient in Pex1 or Pex6 have fewer peroxisomes than wildtype cells, which was eventually attributed to pexophagy [16,17,132]. The mechanism by which Pex1/Pex6 prevents pexophagy is best understood in yeast, where Pex1/Pex6 directly interacts with an autophagy receptor, Atg36, preventing its phosphorylation and activation [18] (Figure 1C, Section 7). Atg36 has no known mammalian homolog, but several pexophagy adaptors have been identified in mammalian cells, including NBR1, p62, and tankyrase [133,134,135]. In mammals, the PEX2-dependent ubiquitination of PMPs is often involved in inducing pexophagy in response to cellular conditions such as nitrogen starvation or oxidative stress [133,136]. Mammalian PEX5 seems to be important for at least some pexophagy pathways [137,138]. For example, the stress-sensing kinase ataxia telangiectasia mutated (ATM) causes pexophagy in response to oxidative stress by phosphorylating PEX5; this triggers mono-ubiquitination at a lysine in the PEX5 N-terminal domain and recruits p62 to the peroxisome membrane [137]. PEX1/PEX6 may normally inhibit this pathway by reducing the amount of PEX5 at the peroxisome membrane. Indeed, separate research found an export-defective variant of PEX5 that accumulates at peroxisomes and triggers pexophagy in some cell lines [138].

Other emerging research supports the notion that Pex1/Pex6 has roles in the cell beyond extracting Pex5 from the peroxisome membrane. Studies in *Arabidopsis* found a suppressor mutant in PEX1 that restored peroxisome function in a PEX6-defective mutant, even though the suppressor did not reduce PEX5 accumulation at the peroxisome [139]. This study suggests a PEX5-independent role for PEX1/PEX6 in peroxisome biogenesis, but it is difficult to rule out a mild increase in PEX5 recycling in these experiments. Others have proposed that PEX1 modulates PEX5 oligomerization in the cytosol [140] and that Pex6 promotes mitochondrial import [141], but these roles are poorly defined. The two N-terminal domains in Pex1/Pex6, rather than the single N-domain in other AAA-ATPases, might allow Pex1/Pex6 to bind a variety of yet unidentified cofactors and substrates. Better characterizing known substrates to establish general requirements for Pex1/Pex6 substrate recognition could help to identify additional endogenous binding partners. 

## 5. Pex1/Pex6 Structure and Threading Mechanism

**Figure 2:**
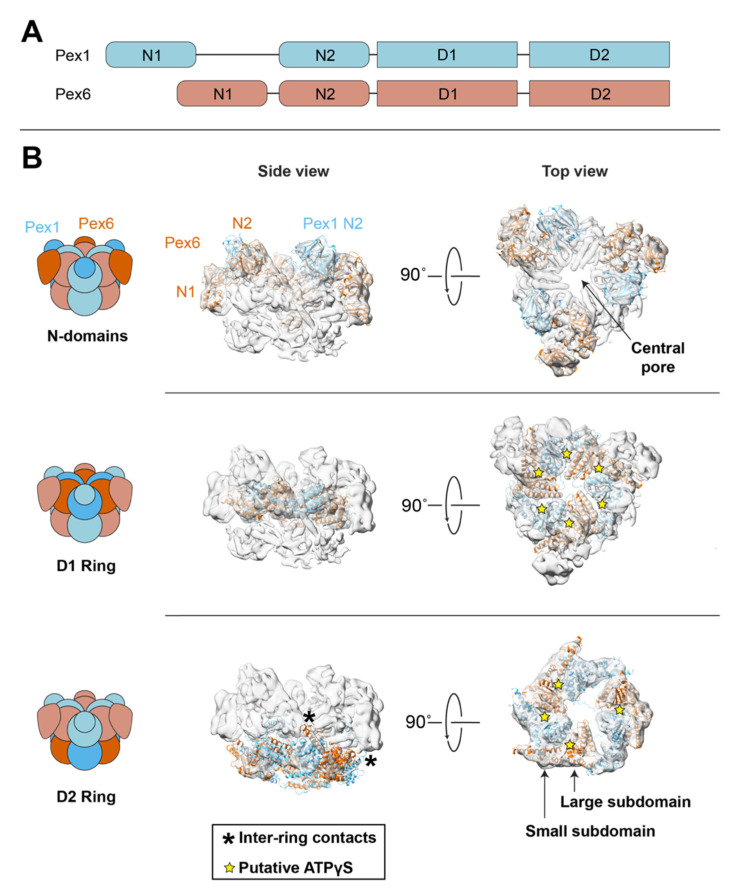
Pex1/Pex6 is a double ring hexameric complex with alternating subunits. (**A**) Pex1 and Pex6 each have two N-terminal domains and two ATPase domains. Pex1 N1 is flexibly attached to the rest of the complex. (**B**) *S. cerevisiae* Pex1/Pex6 structure, based on AlphaFold2 models (red and blue) split by the domain and fitted into EMDB-6359 (gray) [143]. Map resolution: 7.2 Å; fit correlation coefficient: 0.83. The Pex1 and Pex6 N2 domains bind above the D1 ATPase ring, while the Pex6 N1 domain binds to the side of the D1 ATPase ring. The Pex1 N1 domain was not resolved. The D1 ATPase ring binds but does not hydrolyze ATP and is thought to contribute to hexamer assembly. The top view of the active D2 ATPase ring is displayed at a lower threshold than other maps. Asterisks show sites of contact between D1 and D2 rings (see text). Stars represent expected ATP-binding sites based on inter-protomer distances [143].

Here, we discuss Pex1/Pex6’s structure based primarily on *S. cerevisiae* Pex1/Pex6, which has been resolved using negative-stain and cryo-electron microscopy (cryo-EM) [142,143,144]. The models we discuss below for yeast Pex1/Pex6 are composed of AlphaFold2 predictions for each protein [145,146], split into domains, and rigid-body fitted with ChimeraX into a published 7.2 Å cryo-EM map of Pex1/Pex6 [143,147] (Figure 2). AlphaFold2 confidently predicts each domain of Pex1 and Pex6 (Supplementary Figure S1), and the cryo-EM map reveals the relative positions of these domains. Note that in the cryo-EM map, the D2 domain has lower resolution than the rest of the complex, likely reflecting conformational heterogeneity in this ring during the nucleotide hydrolysis cycle. We discuss how the available structures and biochemical data on Pex1/Pex6 integrate with recent advances in understanding substrate processing by other AAA-ATPases. After discussing how the ATPase rings allow Pex1/Pex6 to processively unfold substrates, we turn to the N-terminal domains, which are generally assumed to be involved in cofactor binding and substrate selection. 

### 5.1. Pex1/Pex6 Architecture

**Figure 3:**
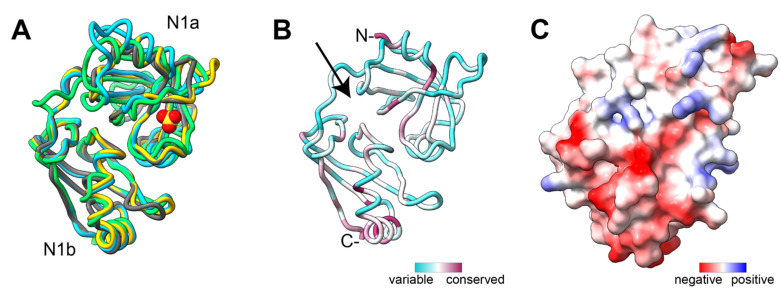
(**A**) The Alphafold2 predictions for Pex1 N1 domain structures in human (yellow), *S. cerevisiae* (green), and *A. thaliana* (blue) aligned to the X-ray crystal structure of murine PEX1 N1 (gray, PDB: 1WLF). The yellow/red ligand represents a sulfate from the PEX1 N1 crystal structure. (**B**) Sequence conservation mapped on the human PEX1 N1 domain. Cofactors such as Npl4 and FAF1 bind a similar cleft between subdomains (arrow) in Cdc48-N. (**C**) Coulombic potential mapped on the surface of human PEX1 N1 domain showing negative charge in the conserved region.

Pex1 and Pex6 assemble into a single hexamer with alternating subunits of Pex1 and Pex6 [142,143,144] (Figure 2). Both Pex1 and Pex6 have two N-terminal domains (N1 and N2) and two ATPase domains (D1 and D2). The ATPase domains hexamerize into two stacked ATPase rings around a central pore (Figure 2). The Pex1 and Pex6 N2 domains are above the D1 ring, while the Pex6 N1 domain is equatorial to the D1 ring. The Pex1 N1 domain is not resolved in electron microscopy structures of Pex1/Pex6 [142,143,144], likely because it is flexibly tethered to the complex. However, the murine PEX1 N1 domain has been separately purified and characterized using X-ray crystallography [148] (Figure 3). 

The canonical motifs required for ATP binding and hydrolysis in AAA-ATPase motors are well characterized (for reviews of AAA-ATPases, see [149,150]). Each ATPase domain consists of two subdomains: a βαβ sandwich and an α-helical bundle, called the large and small subdomains, respectively. In the assembled hexamer, both subdomains contact the large subdomain of the clockwise neighboring subunit (Figure 2), with the small subdomain moving as a rigid body with the neighboring large subunit [151]. ATP binds at the interface between the large and small subdomains and the large subdomain of a neighboring subunit. ATP binding requires a Walker A motif in the large subdomain [152]. Hydrolyzing ATP requires a Walker B motif in the large subdomain, as well as a trans-acting “arginine finger” from the adjacent ATPase domain. To bind substrates in the central pore, each large subdomain extends a pore loop containing an aromatic hydrophobic dipeptide motif that contacts the substrate backbone. For Pex1 and Pex6, both the D1 and D2 have the canonical overall structure for AAA-ATPase domains; however, only the D2 ring preserves the Walker B and arginine finger motifs necessary for ATP hydrolysis and the aromatic pore loops required to grip substrates, indicating that the D2 ring is the active force-generating ring (Table 1). Indeed, mutating Pex1 and Pex6 Walker B motifs in the D2 ATPase abolishes ATPase activity for the complex [142,143,144]. The close proximity of the Pex1 and Pex6 D1 ATPase domains in the cryo-EM structure [143] and conservation of the key lysine residue in the Walker A motif suggest that the D1 ATPase domain maintains nucleotide binding, which assists complex formation. For AAA-ATPases with two rings, it is common for one ring to primarily generate mechanical force, while the other ring mediates assembly and/or selects substrates [153,154].

### 5.2. Pex1/Pex6 Substrate Processing

AAA-ATPases are known to process protein substrates by one of two mechanisms: hydrolysis-driven movements of the N-terminal domains that remodel bound substrates and release them, or hydrolysis-driven movements of the central pore loops that pull substrates through the central pore to unfold them. NSF is the model AAA-ATPase for remodeling substrates by large movements of the N-terminal domains [156], while Clp and proteasomal AAA-ATPases, where the AAA-ATPase translocates substrates into a chambered protease, are the best studied systems for processive treading [149,150]. The majority of unfoldases process substrates by processive threading, even when N-terminal domains are observed to move in a nucleotide-dependent manner [157]. Thus far, all evidence shows that Pex1/Pex6 unfolds client substrates by processive threading; electron microscopy revealed no movements of the N-terminal domains in the presence of various nucleotides (ATP, ATPγS, ADP, or ADP-AlFx) [142,143,144]. Additionally, in vitro studies with Pex1/Pex6 and a model substrate—the cytosolic domain of Pex15 (cytoPex15)—revealed that Pex1/Pex6 uses aromatic central pore loops in the D2 ring to globally unfold cytoPex15 [158]. Once Pex1/Pex6 engaged a flexible C-terminal tail on cytoPex15, it unfolded both Pex15’s core α-helical domain and a flexibly tethered maltose-binding protein attached to Pex15’s N-terminus, confirming that Pex1/Pex6 can move processively along substrates [158].

Although no structure exists for substrate-engaged Pex1/Pex6, high-resolution structures of other AAA-ATPases have recently generated a sequential, hand-over-hand model for how these motors convert ATP hydrolysis into unfolding power [149,150,159,160,161,162,163,164,165,166]. ATPase domains in the active ring typically form a right-handed spiral staircase around the substrate wherein most subunits contact the substrate backbone through aromatic and hydrophobic residues in the pore loops. Subunits at the top of the staircase are typically ATP-bound. ATP hydrolysis is thought to occur in the lower subunits, with phosphate release and opening of the ATP-binding pocket triggering dissociation of the pore loop from the substrate. The empty ATPase subunit is then able to re-bind ATP at the top of the staircase, where the pore loop re-associates with the substrate, thereby pulling the substrate through the central pore. Since a pore loop is bound every two residues, this model predicts that the motor translocates two residues for every ATP hydrolysis. Note that most published high-resolution structures of substrate-bound motors used either slowly hydrolyzable ATP analogues (e.g., ATPγS) or mutations in Walker B motifs that slow hydrolysis and reduce conformational heterogeneity, causing the ATPase domains to favor an ATP and substrate-bound conformation. These conditions may enforce the observed spiral staircase and the sequential hydrolysis typically observed in AAA-ATPases.

How well does Pex1/Pex6 fit this model? The highest-resolution structure of Pex1/Pex6, purified with ATPγS, does not show a spiral staircase in the D2 ring [143] (EMDB-6359, Figure 2). Instead, four of the subunits are planar, while a pair of Pex1/Pex6 rotates downward. The split between this pair of ATPase domains and the rest of the ring occurs at Pex1 ATP-binding sites, such that all three of the Pex6 ATPase domains and only one Pex1 ATPase domain has the inter-subunit spacing to support ATP binding (Figure 2). Note that for some AAA-ATPases, the motor only adopts a spiral staircase when a substrate is bound, so existing structures of Pex1/Pex6 may not reflect the structure of the active motor [157,160,167]. Although the Pex1/Pex6 D2 ATPase has not been observed to form a spiral staircase, the motifs thought to coordinate hydrolysis between subunits in a spiral staircase, namely the intersubunit signaling (ISS) motif and arginine fingers, are conserved in both Pex1 and Pex6 D2 ATPase domains (Table 1). Given the prevalence of the spiral staircase in cryo-EM structures of AAA-ATPases, we expect that the substrate-engaged Pex1/Pex6 can also adopt a spiral staircase under similar conditions.

Despite the prevalence of the observed spiral staircase conformation, several pieces of biochemical data for well-studied motors, such as the Clp AAA-ATPases, are difficult to reconcile with the strictly sequential, hand-over-hand model [168,169,170,171,172,173,174]. Similar biochemical observations, such as translocation rates consistent with large step sizes and residual activity of Walker B mutants, have also been observed for Pex1/Pex6 [142,143,144,158]. Substrate-bound AAA-ATPases in a spiral staircase conformation typically bind stretches of 8–10 amino acids, placing pore loops between every two residues in the substrate. Assuming sequential hydrolysis, this spacing predicts a translocation per ATP hydrolysis of two amino acids. In in vitro studies of Pex1/Pex6 with the cytosolic domain of Pex15, Pex1/Pex6 is more ATP-efficient than would be expected from the hand-over-hand model; on average, it unfolds at least seven residues of cytoPex15 for every ATP hydrolyzed [158]. While this rate is inconsistent with the proposed model of sequential hydrolysis, we note that single-molecule studies of ClpA and ClpX show that these motors take 5–8 residue steps. Since ClpA and ClpX have been shown to form spiral staircases in cryo-EM structures [175,176,177], others have suggested alternative models of probabilistic ATP hydrolysis to account for these larger step sizes [168,169,177,178]. The high efficiency of Pex1/Pex6 can also be explained by the possibilities that multiple substrate chains are translocated simultaneously or that substrate refolding contributes to the translocation rate by preventing backsliding, both of which occur in other motors [179,180]. 

Another prediction of a strictly sequential ATPase model is that inactivating Walker B mutations in either Pex1 or Pex6 should abolish motor activity. While a Walker B mutation in Pex6 D2 does indeed abolish ATPase activity for the entire motor, a Walker B mutation in Pex1 D2 does not eliminate the basal or substrate-engaged ATPase activity in the Pex6 D2 domains of the same ring [142,143] and does not abrogate Pex1/Pex6 function in vivo. These observations suggest that Pex1’s ATPase activity is strictly coordinated with Pex6, such that Pex1 cannot hydrolyze ATP when Pex6 is in an ATP-bound state, while Pex6’s ATPase activity is somewhat independent of Pex1. Residual ATPase activity in Walker B mutants has also been observed for the heterohexameric AAA-ATPase Yta10/Yta12—where Walker B mutations in Yta12 abolish ATPase activity, while Walker B mutations in Yta10 retain 30% of ATPase activity [172]—and for ClpX, which retains ATPase activity when up to four of the six subunits in a hexamer cannot hydrolyze ATP [168]. These inconsistencies suggest the need for alternative models for ATP hydrolysis able to overcome ATP-bound inactive subunits in addition to the strictly sequential, hand-over-hand model.

The lack of coordination of Pex6 with Pex1 predicts that Pex1/Pex6 should have relatively little unfolding power, as coordination between ATPase domains is generally required for maximum unfolding force. For example, ClpX’s unfolding power declines with reduced ATP concentration, suggesting that coordinated hydrolysis in multiple subunits is required to unfold stably folded domains such as GFP [181]. Indeed, Pex1/Pex6 appears to be a weaker unfoldase than some of its homologs; Pex1/Pex6 cannot unfold GFP or methotrexate-bound dihydrofolate reductase (DHFR) [98,138]. Surprisingly, the Pex1 D2 Walker B mutant, which cannot unfold cytoPex15 in vitro, is tolerated in vivo, suggesting that Pex1/Pex6’s role in peroxisome biogenesis does not require the generation of much unfolding force [158]. However, the Pex1 D2 Walker B motif is highly conserved (Table 1), so there may be special circumstances when coordinated hydrolysis and improved unfolding power are important for Pex1/Pex6 function.

In conclusion, while it is possible that Pex1/Pex6 forms a spiral staircase when engaged with substrates, it is difficult to reconcile a fully sequential model of ATPase hydrolysis with the available biochemistry of Pex1/Pex6 nucleotide hydrolysis and substrate processing. Further structural and biochemical work with substrate-bound Pex1/Pex6 is required to explain Pex1/Pex6’s unexpectedly high efficiency and poor ATPase coordination.

### 5.3. Coordination between ATPase Rings

Several other interesting features arise when comparing Pex1/Pex6 to other ATPases. First, electron microscopy of the Pex1/Pex6 complex revealed unexpected contacts between the D1 and D2 ATPase rings mediated by the small ATPase subdomains in the D2 rings. In both Pex1 and Pex6 D2 ATPase domains, an α-helix protrudes from the helical bundles of the small subdomains (Figure 2). In some conformations, the Pex6 D2 helix contacts the Pex1 D1 domain and a disordered loop extending from the Pex1 D2 helix contacts the Pex6 N1 domain [142,143,144,182]. The significance of these contacts is unclear, but they might mediate assembly or coordinate D2 ring activity with substrate binding at the N and D1 domains.

### 5.4. Domains for Substrate Engagement

**Figure 4:**
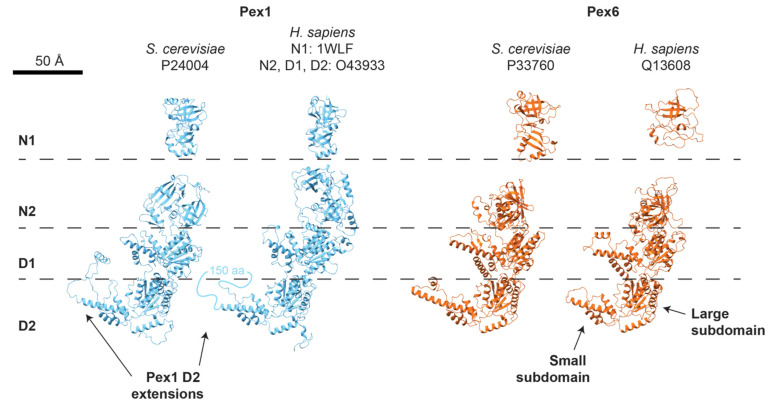
Structures of Pex1 (blue) and Pex6 (orange) monomers from AlphaFold2 or, for *Hs*PEX1 N1, X-ray crystallography (PDB 1WLF, [148]). N1 domains are separated for clarity; Pex1 N1 domains are flexibly attached to the motor, while Pex6 N1 domains are rigidly attached to the Pex6 D1 ring (see Figure 2).

While Pex1/Pex6 hydrolysis and substrate processing is mediated by the active D2 ATPase domains, the selection of substrates most likely depends on the N-terminal and D1 domains. Pex1 and Pex6 each have two N-terminal domains that are structurally similar to the single N-terminal domains in other ATPases, such as NSF and Cdc48, and contain two subdomains: a double-ψ β barrel and an α/β roll [159,183]. The Pex1 N1 domain is the best conserved of the Pex1 and Pex6 N-terminal domains (25% identity between *S. cerevisiae* and *H. sapiens*). The structure of the murine PEX1 N1 has been resolved by X-ray crystallography [148] and comparison of Alphafold2 structural predictions suggests strong conservation of the Pex1 N1 domain fold (Figure 3). The Pex1 N2 domain and the Pex6 N-domains, which are stably bound to the D1 ring, were mapped by structural modeling into cryo-EM density (Figure 2) and are more structurally divergent than Pex1 N1 (Figure 4). The stable position of the N domains relative to the D1 ATPase ring and inter-subunit contacts between the Pex1 and Pex6 N2 domains suggest that the N-domains may play a role in assembling and stabilizing the Pex1/Pex6 heterohexamer around the ATPase-dead D1 ring.

Given that the N-domains in NSF, p97, ClpB, and Lon protease bind adaptor proteins and substrates [156,184,185,186], it is reasonable to expect that the N-terminal domains of Pex1/Pex6 also mediate substrate and cofactor binding. Pex6’s N-domains directly interact with Pex15 in yeast or PEX26 in humans to recruit the hexamer to the peroxisome membrane [11,84,158,187,188]. In turn, Pex15/PEX26 may recruit Pex1/Pex6 to substrates (Section 6). Additionally, in yeast, Pex6’s N-terminal domains interact with Ubp15, which deubiquitinates Pex5 during extraction [115,116].

No binding partners have yet been found for the Pex1 N1 or N2 domains, despite the relatively high conservation of the Pex1 N1 domain. The most likely binding partner for the Pex1 N1 domain is mono-ubiquitinated Pex5, and indeed a structurally similar N-domain in Cdc48 binds ubiquitin-like folds of its binding partners, Ufd1 and Npl4 [157,184] (Figure 3). It is therefore surprising that the Pex1 N1 domain harbors little sequence similarity to the region of the Cdc48 N-domain that interacts with Ufd1 and Npl4. The best conservation instead occurs at the transition to the flexible tether to the Pex1 N2. 

To engage with the D2 pore loops, substrates must be funneled through the D1 ring’s central pore. While this D1 ring is inactive and does not present traditional aromatic pore loops, there are still substantial surfaces for substrate interaction that may act as selectivity filters for substrates. More work is needed to understand how the N-terminal domains and the D1 pore direct Pex1/Pex6 to its substrates, recruit cofactors, and control the motor’s activity.

### 5.5. Comparison to Human PEX1/PEX6

Although several structures exist for yeast Pex1/Pex6, no structure for human PEX1/PEX6 has been reported. Overall sequence identities between human and yeast Pex1/Pex6 are surprisingly low (27% and 24% for Pex1 and Pex6, respectively), but the core ATPase domains are mostly conserved in sequence (Table 1) and Alphafold2 predicts a similar supradomain architecture (Figure 4). As in yeast, human PEX1 and PEX6 are thought to adopt a heterohexameric arrangement at the peroxisome and during receptor recycling [11]. Similar to yeast Pex1/Pex6, the D1 ring of human PEX1/PEX6 does not have the conserved motifs required for ATP hydrolysis (Table 1), and matrix protein import strictly requires ATP hydrolysis only in the PEX6 D2 domain [11]. Previous work predicted that mammalian PEX1 had a C-terminal domain not present in yeast [189]. More recent structure predictions, along with sequence alignments to yeast Pex1, instead predict that the unstructured loop extending from the PEX1 D2 small subdomain, seen in yeast to contact the Pex6 N1 domain, is substantially lengthened in human PEX1 (Figure 4), and that human PEX1 has the same four domains as yeast Pex1. 

Given the same supradomain architecture of the ATPase, substrates for human PEX1/PEX6 are again predicted to engage first with the N-terminal domains and pass through the D1 central pore in order to engage with the D2 pore loops in the active ATPase ring. Of all the putative substrate-binding domains, the PEX1 N1 domain is the best conserved, suggesting that it binds a common conserved substrate. The PEX1 N1 domain is attached to the PEX1 N2 domain by a flexible tether of increased length compared to yeast Pex1. The PEX1 N2 domains have similar interactions with the D1 that predict a conformation above the D1 ring, similar to that observed in yeast. In yeast, the N2 domains interact through strongly charged loops, likely contributing to Pex1/Pex6 assembly. These charge complementarities are less clear in human PEX1/PEX6, indicating that these N2 domains may contribute less to PEX1/PEX6 assembly. The AlphaFold2 predictions for the PEX6 N-domains are generally less robust than for PEX1, particularly for predicted interactions with the D1 ATPase ring (Supplementary Figure S1). However, each of these domains take on a similar fold, which may be stabilized in vivo by binding partners such as PEX26. 

## 6. Pex15/PEX26 as a Partner and Substrate

In *S. cerevisiae*, Pex1/Pex6 is recruited to the peroxisome membrane by the tail-anchored protein Pex15, and this interaction is required to support Pex1/Pex6’s role in matrix protein import and to interact with Atg36 [18,158,190]. Despite the importance of Pex15 in Pex1/Pex6 function, it is remarkably poorly conserved, even in related fungi. However, Metazoa, plants, and other fungi have a functional ortholog, PEX26 (also known as APEM9 in *Arabidopsis*) that is well conserved [12,13,191]. Pex15 or PEX26 is an essential partner for Pex1/Pex6; Pex15/PEX26-deficient cells cannot sustain matrix protein import and accumulate ubiquitinated Pex5 at the peroxisome membrane [103,190,192]. While Pex15 and PEX26 were originally identified as membrane anchors for Pex1/Pex6, subsequent studies, discussed below, raised the possibility that they have more active roles in recruiting Pex1/Pex6 to specific substrates and may even be substrates themselves [18,142,158,193,194,195].

**Figure 5:**
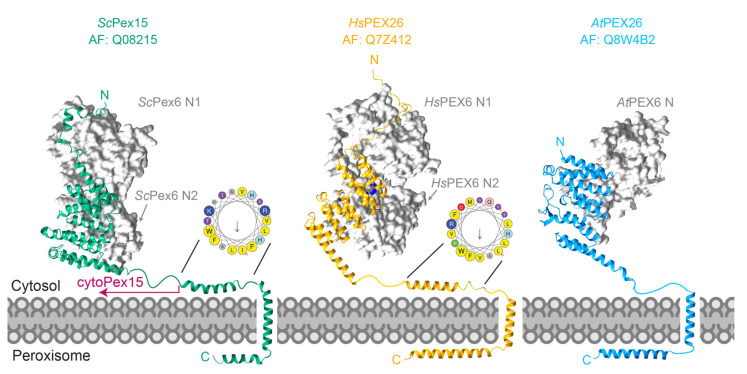
Structural models for the Pex1/Pex6 peroxisomal tethers: *S. cerevisiae* Pex15, *H. sapiens* PEX26, and *A. thalania* PEX26, based AlphaFold2 multimer predictions. Pathogenic missense mutations in PEX26 (D43H, L44P, L45P, G89R, R98W, P117L, and P118R) are colored in blue (Leiden Open Variation Database 3.0, [199]). Predictions for transmembrane domains are from the highest scores from TMHMM-2.0 [200]. Helical wheels generated in Heliquest [201], letters indicate predicted protein sequence.

The structural analysis of *S. cerevisiae* Pex15 using proteolysis and X-ray crystallography revealed that a cytosolic disordered N-terminus precedes a core α-helical domain and a C-terminal transmembrane domain, which is flanked by two membrane-proximal α-helices (Figure 5) [158]. Despite low sequence identity, predictions made using Alphafold2 suggest that Pex15 and PEX26 share this common structure of a cytosolic α-helical core domain followed by a linker to the transmembrane domain (Figure 5). Negative-stain electron microscopy of the yeast Pex1/Pex6/cytoPex15 complex showed densities above the Pex6 N-terminal domains not present in structures of Pex1/Pex6 alone, consistent with an interaction between Pex15 and the Pex6 N-domains identified by yeast 2-hybrid studies and pull-downs [158,187]. The disordered N-terminus of Pex15 was not visible in negative-stain electron microscopy, but the structure is consistent with AlphaFold2-multimer models that predict Pex15’s N-terminus interacting with the Pex6 N1 domain, while the C-terminus extends above the central pore [196] (Figure 5). In humans, PEX26 also binds PEX6 [197], and AlphaFold2 again predicts that PEX26’s core α-helical domain binds PEX6 N2, while an intrinsically disordered N-terminal tail binds PEX6 N1 between the two N1 subdomains [196] (Figure 5). The region of PEX26 predicted to bind PEX6 N1, residues 1-77, is sufficient for binding to PEX1/PEX6 [140]. *Arabidopsis* PEX26 lacks this disordered tail, consistent with *At*PEX6 only having a single N-domain. Further experimental verification is needed to characterize the interaction between PEX6 and PEX26. Both *Sc*Pex15 and *Hs*PEX26 also share an amphipathic helix prior to the transmembrane domain that, for PEX26, has been proposed to mediate homo-oligomerization and binding to other PMPs [194,195]. As an amphipathic helix, it may also deform the peroxisome membrane. The region from the transmembrane domain to the C-terminus is important for binding to Pex19 and peroxisome targeting [198].

After recombinantly expressing yeast Pex1/Pex6 and the cytosolic portion of Pex15 (cytoPex15, Figure 5), Gardner et al. found that Pex1/Pex6 engages cytoPex15’s unfolded C-terminal tail and threads the full protein through its central pore [158], unfolding both Pex15’s core domain and a maltose-binding protein fused to its N-terminus. When cytoPex15 was further truncated from the C-terminus to eliminate the disordered tail, Pex15 was not unfolded. Since modeling of Pex15’s core domain in negative-stain electron microscopy density indicated that this disordered tail is likely positioned above Pex1/Pex6’s central pore, these data suggest that unfolding a substrate requires both binding to Pex6 and a sufficiently long disordered region to engage the Pex1/Pex6 D2 pore loops. In cells, the disordered region of Pex15 that is engaged in vitro is attached to the transmembrane domain (Figure 5); therefore, although this discovery helped establish Pex1/Pex6’s mechanism of unfolding substrates, it is not clear whether Pex15 is a Pex1/Pex6 substrate in vivo. Certainly, unfolding Pex15 is not Pex1/Pex6’s primary contribution to peroxisome biogenesis, since a Pex1 D2 Walker B mutant cannot unfold Pex15 in vitro but supports peroxisome biogenesis, indicating that global unfolding of Pex15 is not essential for peroxisome biogenesis [158]. However, it is also possible that Pex1/Pex6 binds a loop between Pex15’s globular domain and its transmembrane domain without unfolding Pex15. This interaction may help the motor conserve energy, since Pex15 engagement in the central pore inhibits Pex1/Pex6 ATPase activity by up to 80% in vitro [142], in contrast to other AAA-ATPases that increase activity upon binding substrates [202]. In support of a model that Pex1/Pex6 engages Pex15 in vivo, binding between Pex1/Pex6 and Pex15 is enhanced in a Pex6 D2 Walker B mutant, a mutation known to trap substrates in the central pore of AAA-ATPases [188,203].

Other AAA-ATPases use their N-domains to bind cofactors, which subsequently recruit substrates. Attached to the peroxisome membrane and bound to the Pex6 N-domains, Pex15/PEX26 is well positioned to bind substrates and direct Pex1/Pex6 activity. For example, Pex15 recruits Pex1/Pex6 to one substrate, Atg36, by binding Atg36’s peroxisome membrane receptor Pex3 [18]. Pex15 and PEX26 also strongly interact with the DTM by binding Pex14 and Pex5 [84,140], and Pex15 enables in vitro binding between Pex5/Pex14 and Pex1/Pex6 [158]. Notably, both faces of the PEX26 core domain—the predicted PEX6-binding face and the strongly acidic opposing side—are relatively well conserved, suggesting that PEX26 may have an additional binding partner besides PEX6. Most pathogenic missense mutations in PEX26 are in this core domain and seem to reduce folding efficiency [12] (Figure 5). Further mutational analysis of PEX26 could help distinguish residues important for protein folding, interaction with PEX6, and interaction with potential substrates. 

Additional evidence that PEX26 plays a role beyond anchoring PEX1/PEX6 to the peroxisome membrane arose from the observation that human cells express both full-length tail-anchored PEX26 and a splice variant lacking the transmembrane domain. Surprisingly, both isoforms are sufficient to support peroxisome biogenesis [193,195]. Furthermore, artificially targeting PEX26 to mitochondria by replacing the PEX26 transmembrane domain with a mitochondrial tail-anchoring sequence redirects PEX1/PEX6 to mitochondria, but still supports peroxisome biogenesis [193]. These findings suggest that PEX26 does not need to localize to the peroxisome, contradicting the belief that PEX26’s primary role is to recruit PEX1/PEX6 to the peroxisome membrane. Although suggestive, these results should be interpreted with caution because some PEX26 may have localized to peroxisomes in both experiments; a fraction of spliced soluble PEX26 binds to PEX14 at the peroxisome, and mitochondrial tail-anchored proteins are known to mistarget to the peroxisome [195,204]. Nevertheless, the phenotypic similarity between PEX26 mutants and PEX1/PEX6 mutants [193,197,205] and the increasing evidence that PEX26 is more than a membrane tether warrant further investigation into the role of PEX26 in peroxisome biogenesis.

## 7. Potential Substrates of Pex1/Pex6

**Figure 6:**
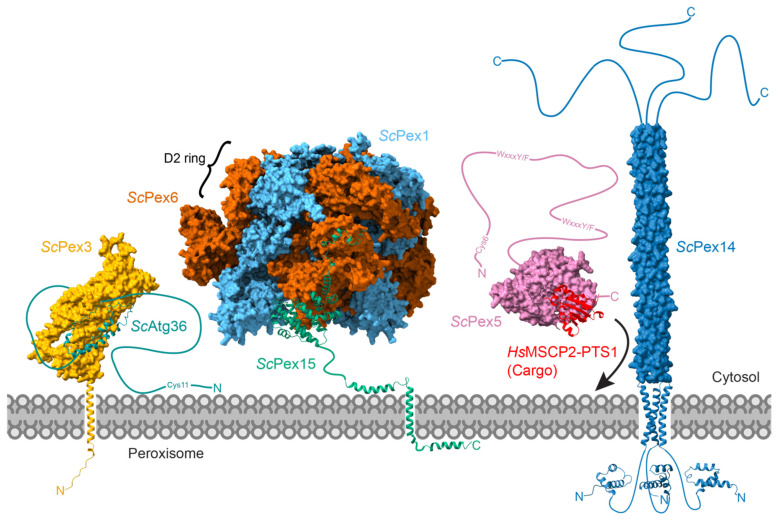
Putative Pex1/Pex6 substrates (cytoPex15, Pex5, and Atg36) have disordered tails (curved lines) capable of accessing the Pex1/Pex6 D2 pore. Pex1/Pex6 is recruited to the peroxisome membrane by Pex15 (models based on EMDB-6359; PDB 5VXV; Alphafold2 multimer). In vitro, the truncated cytosolic domain of Pex15 is a Pex1/Pex6 substrate. Pex5 bound to a PTS1-tagged protein (model based on *Hs*PEX5 bound to MSCP2 PDB: 2C0L; Alphafold2 *Sc*Pex5 AF: P35056) embeds in the DTM, primarily composed of Pex5 bound to Pex14 (modeled from EMDB-12047 [10.2 Å]; Alphafold2 AF: P53122). Pex1/Pex6 is then thought to extract mono-ubiquitinated Pex5 from the membrane. Atg36 interacts with Pex1/Pex6 indirectly through Pex3 and Pex15 (models based on Alphafold2 multimer). Pex1/Pex6 prevents Atg36 phosphorylation, though the mechanism is unclear. Figures made with ChimeraX 1.3 [147].

The identity of substrates that Pex1/Pex6 acts on is still an active area of research, but a few probable substrates have emerged in recent years (Figure 6). Pex5 and the PTS2 co-receptors, Pex18, Pex20, and Pex21, are generally considered the most important in vivo substrates, but researchers have yet to show that these are directly unfolded by Pex1/Pex6. As discussed above, Pex15 and PEX26 are additional possible substrates, based primarily on in vitro experiments with Pex15 and yeast Pex1/Pex6 [142,158]. Finally, Pex1/Pex6 was recently reported to directly interact with the yeast autophagy receptor Atg36 to prevent its phosphorylation [18]. From these observations, we can begin to infer general requirements for Pex1/Pex6’s substrates.

Pex5 has long been thought to be the relevant substrate for Pex1/Pex6 at the peroxisome membrane [102]. As discussed above, Pex5 binds PTS1-labeled proteins in the cytosol, and through interactions with the DTM, including Pex14, becomes embedded in the peroxisome membrane during import [15,65,102,206] (Figure 6). To conduct additional rounds of import, Pex5 is mono-ubiquitinated and extracted from the membrane in a process reminiscent of Cdc48-dependent ER-associated degradation [15,102,103,207]. Extraction requires ATP and catalytically active assembled Pex1/Pex6 [102,192]. Recombinant Pex1 directly binds Pex5 (K_D_ = ~1.0 μM), an interaction that is improved by Pex5 mono-ubiquitination [140,208,209]. Additionally, in cell-free in vitro experiments with rat liver homogenate, researchers showed that buried cysteines in PEX5’s PTS1-binding domain become solvent-exposed during extraction [98]. Such unfolding is required for extraction: a stably folded domain at the C-terminus of Pex5 (either replacing or supplementing the PTS1-binding domain) blocks peroxisome biogenesis and extraction [98,138]. Together, these experiments support the hypothesis that Pex1/Pex6 directly extracts Pex5 from the peroxisome membrane. Nevertheless, researchers have yet to reconstitute Pex1/Pex6-mediated unfolding of Pex5 from purified components, indicating that additional requirements for engagement of Pex5 remain to be described. 

How might Pex1/Pex6 engage ubiquitinated Pex5? As described above, Pex5 at the peroxisome membrane is embedded in the DTM and resistant to protease digestion [82,206], but a conserved N-terminal cysteine (*S. cerevisiae*: Cys6, *H. sapiens*: Cys11) is exposed to the cytosol to be mono-ubiquitinated by the membrane-bound RING finger complex Pex2/Pex10/Pex12 (E3) and a cytosolic E2, either Pex4 in yeast or UBCH5 in humans. It is reasonable to suppose that Pex1/Pex6 binding to Pex5 requires interactions with both ubiquitin and with a disordered loop of Pex5 that can reach the Pex1/Pex6 D2 pore loops. Ubiquitinated Pex5 has been shown to interact with Pex1 with higher affinity than unmodified Pex1, suggesting that Pex1 has a binding site for ubiquitin [208,209]. It has also been shown that ubiquitin can crosslink to both Pex1 and Pex6 [98], but a clear binding site for ubiquitin has yet to be established. Pex5 binding may be mediated by a cofactor, such as Pex15/PEX26 or the mammalian ubiquitin-binding protein AWP1, which binds both PEX6 and ubiquitinated PEX5 and promotes PEX5 recycling and peroxisome import [210]. Pex1/Pex6 engagement with Pex5 could also require DTM components or the peroxisome membrane.

Although the extreme N-terminus of Pex5 is available to cytosolic Pex1/Pex6, it is unlikely that the motor begins by engaging this N-terminal tail for several reasons. The ~4-10 residues from the N-terminal methionine to the cysteine-linked ubiquitin are too short to thread through the D1 ring and engage D2 pore loops. Additionally, linear ubiquitin-Pex5 fusions—wherein Pex5 does not have an N-terminal tail—are exported similarly to native Pex5 [98]. Finally, N-terminally fusing eGFP to Pex5 does not substantially impair peroxisome biogenesis [138]. Three alternative models for Pex5 engagement using Pex1/Pex6 remain [211]. In the first model, Pex1 might start by unfolding the attached ubiquitin, as is the case for Cdc48/p97 [212]. In this model, Pex1/Pex6 must be able to process two polypeptides simultaneously upon reaching the ubiquitin attachment site, but other AAA-ATPases have been shown to thread protein loops [180,212,213]. Secondly, Pex1/Pex6 could engage a disordered loop in Pex5’s N-terminal domain downstream of the ubiquitin attachment site. Once again, this mechanism requires Pex1/Pex6 to engage a loop. Finally, Pex1/Pex6 might assemble around ubiquitinated Pex5, between the attached ubiquitin and the C-terminal domain, much like Vps4 assembly around a disordered region in its substrates [214]. According to this final model, Pex1/Pex6 needs to be a dynamic complex capable of readily disassembling and reassembling. A key component of these models is that Pex1/Pex6 should only bind and process Pex5 when ubiquitinated and embedded in the peroxisome membrane, since unfolding in the cytosol and release of PTS1-tagged cargo might be counterproductive for peroxisome targeting. 

While Pex5 is the best-studied potential substrate of Pex1/Pex6, the N-terminal domains of the PTS2 cofactors likely engage with Pex1/Pex6 in a similar fashion. In yeast, these include the newly discovered Pex9, which is partially redundant with Pex5 but under separate regulation, and the PTS2 co-receptors Pex18 and Pex21 (Pex20 in some fungi) [62,91,215,216]. All these proteins contain WxxxF motifs along a disordered N-terminal domain and a conserved cysteine near the N-terminus, and Pex18 and Pex20 have been shown to be monoubiquitinated [99,100]. In mammals, the longer spliced version of PEX5, PEX5L, is expected to have the same N-terminus and therefore the same modifications and motor engagement as PEX5.

Recently, Yu and colleagues identified another possible substrate for Pex1/Pex6, the autophagy receptor Atg36, an intrinsically disordered protein that binds Pex3 [18] (Figure 6). During pexophagy, Atg36 is phosphorylated by the cytosolic kinase Hrr25 [217], which triggers binding of Atg11 and the Atg1 complex and autophagosome formation. Pex1/Pex6 inhibits Hrr25-dependent phosphorylation, thus preventing pexophagy. Yu et al. did not identify a direct mechanism for Pex1/Pex6 preventing autophagy, but Atg36 has many of the features of the other putative Pex1/Pex6 substrates discussed above. Like Pex5 and the PTS2 co-receptors, Atg36 harbors an N-terminal cysteine, although this residue is not known to be ubiquitinated and its significance is unclear. Atg36 is also predicted to be an intrinsically disordered protein, and therefore likely contains unstructured regions for Pex1/Pex6 engagement. Furthermore, Atg36 binds Pex3, which recruits Pex1/Pex6 by binding Pex15 [18,218]. Note that the mechanisms for pexophagy regulation are not conserved between yeast and mammals [50], so these findings do not indicate the existence of a PEX5-independent function for PEX1/PEX6 in humans. Nonetheless, they suggest that Pex1/Pex6 can act on multiple substrates and has multiple roles in peroxisome homeostasis.

From these substrates, as well as the in vitro characterization of Pex15 unfolding by Pex1/Pex6, a few general requirements emerge for Pex1/Pex6 substrates. Any substrate must bind to Pex1/Pex6 either directly (e.g., Pex15) or indirectly (e.g., Atg36). Mono-ubiquitination may increase substrate affinity for Pex1/Pex6 [208,209]. Substrates must have an unstructured tail or loop of at least 30-40 residues that can pass through the Pex1/Pex6 inactive D1 ring and engage with D2 pore loops to begin unfolding [158]. Pex5, Atg36, and truncated Pex15 all meet these requirements (Figure 6). Other AAA-ATPases can engage unstructured loops and process two strands simultaneously [180,212,213], so a Pex1/Pex6 substrate might likewise have an unstructured domain flanked by folded domains. Notably, Pex1/Pex6’s apparent inability to unfold GFP and methotrexate-bound DHFR suggests limited unfolding power compared to other AAA-ATPase motors [98,138].

## 8. Peroxisomes and Pex1/Pex6 in Disease

Mutations in the PEX genes that affect peroxisome formation or the import of peroxisome matrix proteins cause peroxisome biogenesis disorders (PBDs), including Zellweger spectrum disorders and rhizomelic chondrodysplasia punctata [219]. Cells with mutations in the PEX genes may have reduced numbers of peroxisomes, lack peroxisomes entirely, or produce peroxisome “ghosts”, which have PMPs but few or no matrix proteins [220,221,222,223]. The loss of peroxisome function disrupts cellular redox homeostasis and impairs mitochondrial function [3,224]. Clinically, peroxisome dysfunction is associated with reduced levels of peroxisome products, including plasmalogens and docosahexaenoic acid, and an accumulation of fatty acids normally oxidized in the peroxisome, particularly very long-chain fatty acids and phytanic acid [19]. Very long chain fatty acid accumulation and dysfunctional lipid metabolism in the nervous system cause some of the common symptoms of Zellweger spectrum disorders, including demyelination and neurological defects [55,225,226]. PBDs are phenotypically diverse—their symptoms and severity depend on the mutation, genetic background, and environment [226]. 

Mutations in PEX1, PEX6, or PEX26 cause most PBDs (48.5%, 13.1%, and 3.4%, respectively) and represent a wide range of disease severity [19]. Diseases caused by complete loss of function in any of these genes are fatal in utero or during infanthood, but conservative mutations can cause mild phenotypes, such as Heimler’s syndrome [227,228]. Without PEX1, PEX6, and PEX26, PEX5 accumulates at the peroxisome membrane [192]. Additionally, mutations in PEX1, PEX6, and PEX26 are associated with increased pexophagy [16,229], but it is still unclear precisely how defects in PEX1/PEX6/PEX26 cause pexophagy in mammalian cells. As described above, one model is that PEX1/PEX6/PEX26 prevent an accumulation of ubiquitinated PEX5 at the membrane, thereby preventing ubiquitin-mediated pexophagy (Section 4) [133,136,137,138]. A hypothesis briefly arose that pexophagy is the primary cause of disease in patients with mutations in PEX1, PEX6, or PEX26 [6,230]. However, recent findings show that inhibiting pexophagy in PEX1-deficient patient fibroblasts does not restore matrix protein import or improve metabolic function in PBD cells [229], indicating the existence of peroxisome import defects beyond an increased rate of pexophagy. Thus, recycling PEX5 from the peroxisome membrane remains the canonical role for PEX1/PEX6/PEX26 in mammals. More research is needed to determine the mechanism and significance of the complex in preventing pexophagy.

Nearly half of PBD patients have a mutation in PEX1, which is most commonly a missense mutation converting glycine 843 to aspartic acid (G843D) [231]. Other reviews comprehensively discuss pathogenic mutations in PEX1 and PEX6 [189], so we restrict our discussion here to PEX1-G843D. Patients homozygous for PEX1-G843D display milder forms of PBDs and only a partial loss in matrix protein import compared to patients with truncation variants of PEX1, suggesting that PEX1-G843D is a hypomorphic allele [232]. PEX1-G843D is associated with reduced *PEX1* mRNA levels (~50%), and further reduced levels of PEX1 protein (3–20% of wildtype) [232,233]. Even in cases where PEX1-G843D levels are not reduced, such as in mouse models of PEX1-G843D, the mutation still causes symptoms consistent with peroxisome dysfunction, such as vision impairment and altered metabolism, indicating that PEX1-G843D is hypomorphic even at wildtype protein levels [234]. Since PEX1-G843D has reduced association with PEX6 and overexpression of PEX6 can ameliorate peroxisome defects [227], it appears that the primary defect of PEX1-G843D is reduced assembly with PEX6, which is exacerbated by reduced levels of PEX1-G843D. Matrix protein import is only partially lost, so PEX1-G843D/PEX6 must be at least partially active once assembled.

**Figure 7:**
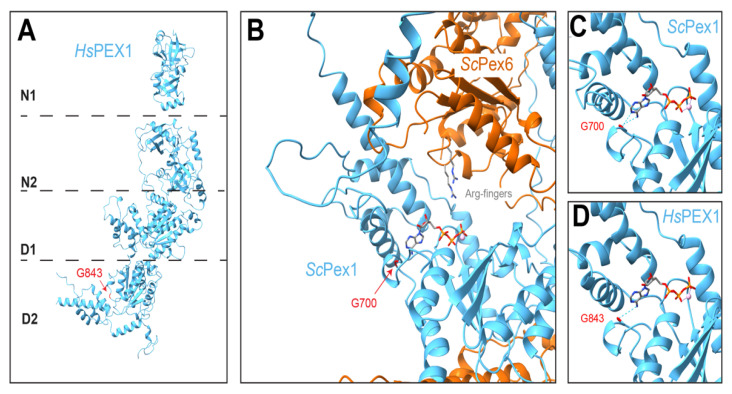
PEX1 G843D is expected to disrupt ATP binding and/or protomer folding. (**A**) *Hs*PEX1, based on AlphaFold2 and X-ray crystallography (as in Figure 2, PDB 1WLF, [148]). G843 is in the PEX1 D2 ring. (**B**) *Sc*Pex1 D2 ATPase in the Pex1/Pex6 hexamer at the Pex1 site most likely to be nucleotide-bound (AlphaFold2, EMDB-6359, [143]). Note that nucleotides are not discernible in experimental structures of Pex1/Pex6; an ATP is modeled based on alignment with a high-resolution structure of *Hs*p97 (PDB 7LN5, [159]). (**C**) The glycine G843 in *Hs*PEX1 is conserved. The homologous residue in *Sc*Pex1 (G700) is colored in red and the backbone is predicted to hydrogen bond with the adenosine of ATP ([159], ChimeraX). (**D**) In the structurally similar *Sc*Pex1 and HsPEX1 ATPase sites, G700 or G843 hydrogen bond (dotted lines) with ATP.

Sequence alignments show that PEX1 Gly843 is conserved in other organisms and in related AAA-ATPases. The glycine is in a loop in the D2 large subdomain that contacts the nearby small subdomain (Figure 7). It is possible that the G843D could disrupt protein folding in this region due to the added bulk of the aspartic acid sidechain or more limited torsion angles allowed for aspartic acid compared to glycine. However, since the Gly843 equivalent in other ATPases contacts ATP [143,235], (Figure 7), the mutation may also preserve PEX1 folding but disrupt ATP binding. ATP binding at the PEX1 D2 ATPase site is known to be important for complex assembly [236], yet ATPase activity in the PEX1 D2 ATPase is dispensable for Pex1 function in vivo [144,158]. Thus, it remains to be tested if PEX1-G843D impacts PEX1/PEX6 activity of the assembled motor. 

Several small molecules are known to improve PTS1 protein import and peroxisome function in PEX1-G843D patient fibroblasts, including chemical chaperones such as betaine, glycerol, and trimethylamine N-oxide. Additionally, a class of flavonoid-based molecules was identified in a screen for small molecules that improve PTS1 protein import in PEX1-G843D patient fibroblasts [237,238]. It remains unclear if these small molecules directly stabilize PEX1, increase assembly with PEX6, or work through some other mechanism, but they emphasize the importance of proteostasis in PEX1-G843D pathology. Despite the high similarity between ATPase domains of various AAA-ATPases, specific inhibitors of p97 have been identified that bind in cryptic binding pockets between ATPase domains with promising antiviral and antitumor activity [159,239,240,241]. These molecules suggest the feasibility of identifying small molecule modulators specific to PEX1/PEX6.

With the ongoing advances in our understanding of Pex1/Pex6 structure and biochemistry, future medical research efforts can focus on directly targeting PEX1-G843D/PEX6. Given the apparent assembly defect in PEX1-G843D and at least partial activity of the assembled motor, a focus of future research efforts is to find ways to promote the interaction between PEX1-G843D and PEX6. Extrapolating from the yeast Pex1/Pex6 structure, PEX1 and PEX6 interact at both the PEX1 and PEX6 ATP-binding sites of the D1 and D2 rings (Figure 2), and assembly at the PEX1 ATPase interface is nearly entirely mediated by interactions between ATPase domains in the D1 and D2 ring, without contributions from the N2 domains above the D1 ring (Figure 2). Assembly is therefore likely to depend on cellular ATP concentration and might be improved by small molecules that bind near the ATPase-binding pockets. Another possible point of contact is between the extended loop in the Pex1 D2 ATPase small subdomain and the Pex6 N1 domain, as revealed by negative-stain and cryo-electron microscopy of yeast Pex1/Pex6 [142,143,144]. Alphafold2 predicts that this loop is extended in human PEX1. This loop is subject to phosphorylation, suggesting it as a possible point of regulation [242,243,244]. A high-resolution structure of the human PEX1/PEX6 is needed to help characterize these interfaces and aid small-molecule design to increase PEX1/PEX6 assembly.

The reduced levels of PEX1-G843D protein likely contribute to the PEX1/PEX6 assembly defect. PEX1-G843D protein levels are almost fully recovered when cells are cultured at 30 °C rather than 37 °C [233], and the increased levels correlate with improved matrix protein import. Thus, pathways that control PEX1 abundance may be therapeutic targets for PEX1-G843D. For example, the RNA-binding protein heterogeneous nuclear ribonucleoprotein A1 (HNRNPA1) binds and stabilizes PEX1 mRNA, increasing its expression and preventing pexophagy [245]. The pathway targeting PEX1-G843D for degradation may also have specific adaptors that could be inhibited. For instance, other AAA-ATPase motors have dedicated assembly factors to facilitate assembly and prevent degradation of unassembled protomers [246,247]. Further research is needed to identify and target mechanisms regulating PEX1 stability and degradation in human cells.

While much of the focus on understanding the role of peroxisomes in human disease is focused on the rare genetic peroxisome biogenesis disorders, there is increasing interest in the impact of peroxisome dysfunction on nonhereditary and age-related diseases. In model organisms such as *C. elegans*, fruit flies, and mice, PEX protein levels decline with age [248,249,250]. Recent studies looking at the induced loss of PEX1 or overexpression of PEX5 mutants incapable of protein extraction [17,251] are starting to provide insight into the impact of peroxisome stress on otherwise healthy cells. The connection between peroxisomes and mitochondria may be particularly relevant for aging phenotypes, as peroxisome dysfunction impairs mitochondrial function [224]. Interestingly, overexpressing Pex6 in yeast can suppress cellular dysfunction associated with mitochondrial aging [141], so improved peroxisome function may also improve mitochondrial function. Mitochondria and peroxisomes also share signaling pathways, such as mitochondrial antiviral signaling protein (MAVS), which contribute to innate immunity [252]. The variety of roles of peroxisomes, including ROS signaling and lipid metabolism, as well as their platform for cell signaling, suggest the importance of investigating peroxisome dysfunction in a variety of cell types.

## 9. Conclusions

The AAA-ATPase Pex1/Pex6 drives protein import into peroxisomes and is thus a key step in peroxisome formation and a common culprit for peroxisome biogenesis disorders. Although recent insights have considerably advanced our understanding of Pex1/Pex6 structure and function, questions remain regarding Pex1/Pex6 substrate selection and processing, the assembly and regulation of human PEX1/PEX6, and the consequences of mutations in human PEX1/PEX6. We expect insights to come from identifying the full repertoire of Pex1/Pex6 substrates, structures of Pex1/Pex6 unfolding a substrate, in vitro reconstitutions of Pex5 recycling to define the minimum components for Pex1/Pex6 activity, structures of human PEX1/PEX6, and further analysis of disease-causing alleles. Peroxisomes are integral to cell metabolism and human health, and employ unique strategies for solving basic problems in cell biology, such as protein translocation and extraction from membranes. Understanding peroxisome matrix protein import will be an important advance in cell biology, with applications in human health and synthetic biology [53,253,254,255].

## Figures and Tables

**Table 1:** Important Motifs for Active AAA-ATPases are Conserved only in Pex1/Pex6 D2 ATPase Domains.

		Walker A	Pore Loop 1	Walker B	ISS	Arg Finger
		ATP Binding	Substrate Binding	ATP Hydrolysis	Inter-Subunit Signaling	Assembly, ATP Hydrolysis
**Consensus**		GxxGxGKT	+ΩΦx-	ΦΦΦΦDE	DGF	ALLRPGR
	*Sc*Pex1	GKQGIGKT	CETLHE-TSNLDKTQ	LIVLDN	QVTKI	LLFDKHF
	*Hs*PEX1	GGKGSGKS	CKALR--GKRLENIQ	VVLLDD	MIKEF	LLV…VHI
	*At*PEX1	GPPGSGKT	CSTLA--LEKVQHIH	VIILDD	VIDDY	TLSSSGR
**D1**	*Sc*Pex6	S…NNVGKA	CLSLTSNSRQLDSTS	VIFLAH	LLDDF	SFRS--H
	*Hs*PEX6	GPPGCGKT	CSSLC--AESSGAVE	VLLLTA	LLLNE	DVQ--TA
	*At*PEX6	GIPGCGKR	CHSLL--ASSERKTS	ILLLRH	VIREL	TIR--RC
	*Sc*Pex1	GYPGCGKT	GPEIL--NKFIGASE	ILFFDE	QMDGA	ALLRPGR
	*Hs*PEX1	GPPGTGKT	GPELL--SKYIGASE	ILFFDE	QLDGV	ALLRPGR
**D2**	*At*PEX1	GPPGCGKT	GPELL--NKYIGASE	ILFFDE	ELDGV	ALLRPGR
	*Sc*Pex6	GPPGTGKT	GPELL--NMYIGESE	VIFFDE	ELDGM	ALLRPGR
	*Hs*PEX6	GPPGTGKT	SPELI--NMYVGQSE	IIFFDE	ELDGL	ALLRPGR
	*At*PEX6	GPPGTGKT	GPELI--NMYIGESE	VIFFDE	EMDGM	ALLRPGR

Structural alignments were generated with MUSTANG [155] based on AlphaFold2 structures. Grey residues match consensus sequences for the given motif [149,150] *Sc*: *Saccharomyces cerevisiae*, *Hs*: *Homo sapiens*, *At*: *Arabidopsis thaliana*.

## Data Availability

Not applicable.

## Supplementary Materials

The following supporting information can be downloaded at: https://www.mdpi.com/article/10.3390/cells11132067/s1. Figure S1: Confidence metrics for AlphaFold2 predictions.
