# Supplementary figures and images for: Insights into the Structure and Function of the Pex1/Pex6 AAA-ATPase in Peroxisome Homeostasis
Source: Cells. 2022 Jun 29;11(13):2067. doi: 10.3390/cells11132067 (PMC9265785; doi:10.3390/cells11132067)

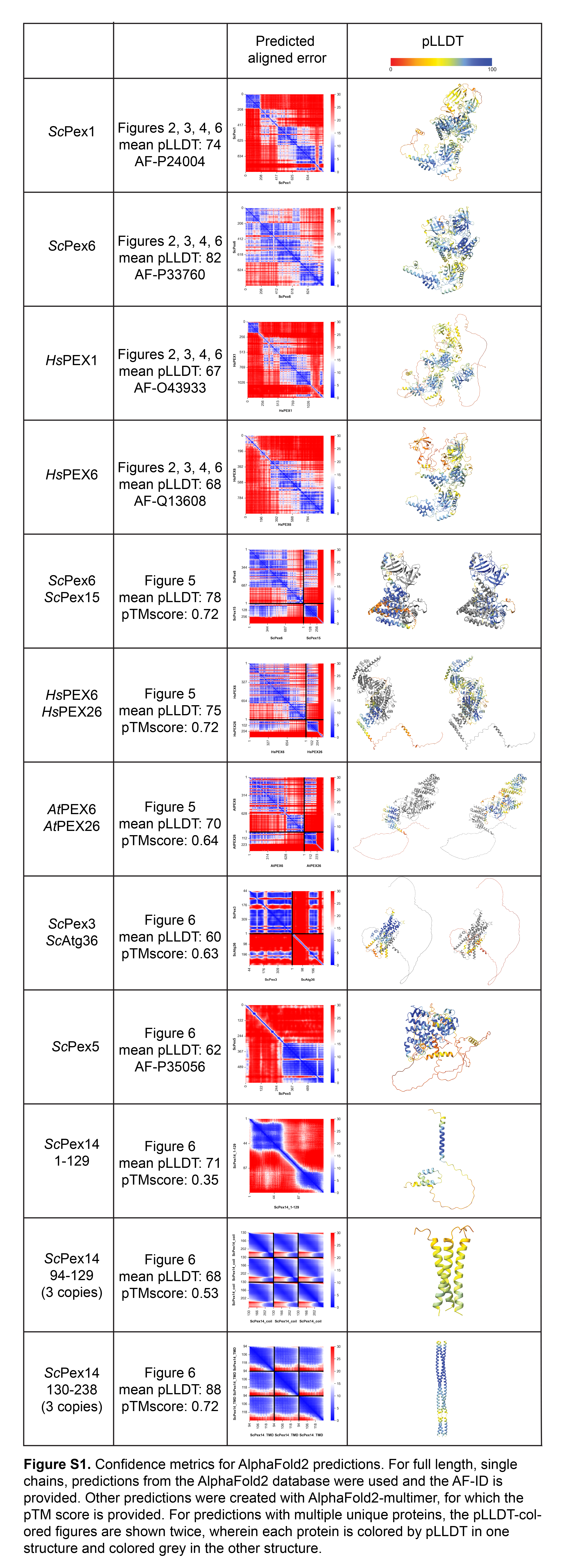

Supplement: Supplementary file 1 [file cells-11-02067-s001.zip › cells-1778518-supplementary.png]
